# Curcumin protects against palmitic acid-induced apoptosis via the inhibition of endoplasmic reticulum stress in testicular Leydig cells

**DOI:** 10.1186/s12958-019-0517-4

**Published:** 2019-08-31

**Authors:** Zhi Chen, Di Wen, Fen Wang, Chunbo Wang, Lei Yang

**Affiliations:** 1 College of Biological Science and Agriculture, Qiannan Normal University for Nationalities, Duyun, 558000 Guizhou China; 2 Key Laboratory of System Bio-medicine of Jiangxi Province, Jiujiang University, Jiujiang, 332000 Jiangxi China; 3 College of Basic Medical Science, Jiujiang University, Jiujiang, 332000 Jiangxi China

**Keywords:** Leydig cell, Palmitic acid, Apoptosis, Curcumin, Endoplasmic reticulum stress

## Abstract

**Background:**

Palmitic acid (PA) is a common saturated fatty acid that induces apoptosis in various types of cells, including testicular Leydig cells. There is evidence suggesting that PA is increased in patients with obesity and that PA-induced cell apoptosis may play an important role in obesity-related male infertility. Curcumin, a natural polyphenol, has been reported to exert cytoprotective effects in various cell types. However, the cytoprotective effect of curcumin against PA-induced apoptosis in Leydig cells remains unknown. Therefore, the current study was performed to investigate the protective effects of curcumin in response to PA-induced toxicity and apoptosis in murine Leydig tumor cell line 1 (MLTC-1) cells and explore the mechanism underlying its anti-apoptotic action.

**Methods:**

MLTC-1 cells were cultured in Roswell Park Institute-1640 medium and divided into five groups. First four groups were treated with 50–400 μM PA, 400 μM PA + 5–40 μM curcumin, 400 μM PA + 500 nM 4-phenylbutyric acid (4-PBA, an endoplasmic reticulum (ER) stress inhibitor), and 500 nM thapsigargin (TG, an ER stress inducer) + 20 μM curcumin, respectively, followed by incubation for 24 h. Effects of PA and/or curcumin on viability, apoptosis, and ER stress in MLTC-1 cells were then determined by cell proliferation assay, flow cytometry, and western blot analysis. The fifth group of MLTC-1 cells was exposed to 400 μM of PA and 5 IU/mL of human chorionic gonadotropin (hCG) for 24 h in the absence and presence of curcumin, followed by measurement of testosterone levels in cell-culture supernatants by enzyme-linked immunosorbent assay (ELISA). Rats fed a high-fat diet (HFD) were treated with or without curcumin for 4 weeks, and the testosterone levels were detected by ELISA.

**Results:**

Exposure to 100–400 μM PA reduced cell viability, activated caspase 3, and enhanced the expression levels of the apoptosis-related protein BCL-2-associated X protein (BAX) and ER stress markers glucose-regulated protein 78 (GRP78) and CCAAT/enhancer binding protein homologous protein (CHOP) in MLTC-1 cells. Treating cells with 500 nM 4-PBA significantly attenuated PA-induced cytotoxicity through inhibition of ER stress. Curcumin (20 μM) significantly suppressed PA- or TG-induced decrease in cell viability, caspase 3 activity, and the expression levels of BAX, CHOP, and GRP78. In addition, treating MLTC-1 cells with 20 μM curcumin effectively restored testosterone levels, which were reduced in response to PA exposure. Similarly, curcumin treatment ameliorated the HFD-induced decrease in serum testosterone level in vivo.

**Conclusions:**

The present study suggests that PA induces apoptosis via ER stress and curcumin ameliorates PA-induced apoptosis by inhibiting ER stress in MLTC-1 cells. This study suggests the application of curcumin as a potential therapeutic agent for the treatment of obesity-related male infertility.

## Background

Obesity is known to be a major risk factor for male infertility, and therefore, obesity-associated male infertility is increasingly drawing public attention [1–4]. So far, the mechanisms underlying obesity-induced male infertility remain unclear. PA is the most common type of saturated free fatty acids (FFAs) in the plasma. It has been reported that FFAs, including PA, are increased in patients with obesity [5–7]. Elevation in the level of FFA, especially the saturated ones including PA, has been suggested to be closely associated with obesity-induced male infertility [5, 6, 8]. An earlier study has demonstrated that PA markedly suppresses cell survival and induces apoptosis in rat testicular Leydig cells in a time- and dose-dependent manner [9]. This suggests that Leydig cell toxicity induced by PA contributes to, or may even cause, reproductive abnormalities in obese men.

ER stress is defined as an imbalance between the protein load and the folding capacity of the ER, resulting in the accumulation of unfolded or misfolded proteins in the ER lumen. Therefore, the ER stress response is also commonly known as the unfolded protein response [10, 11]. ER stress or impaired ER homeostasis has been reported to be closely associated with the pathology of reproductive diseases [12]. In a study on obese mice, ER stress-mediated spermatocyte apoptosis was shown to be enhanced through CHOP and caspase-3 activation [13]. In addition, accumulating evidence suggests that ER stress is activated in various tissues under conditions related to obesity [10]. Excessive ER stress has been shown to ultimately induce cellular apoptosis [14]. Although PA has been implicated to induce apoptosis in rat testicular Leydig cells, the involvement of ER stress in this process remains unknown [9].

Curcumin is a phytochemical component isolated from turmeric (*Curcuma longa* L., Zingiberaceae), and because of its anti-oxidant, anti-inflammatory, and anti-obesity activities, it has been widely used in studies on infertility and metabolic disorders, including obesity [15–19]. Curcumin has been reported to effectively attenuate ER stress-induced cell apoptosis in various cell types [20–22]. Nevertheless, it is still unclear whether curcumin exhibits protective effects through inhibition of ER stress against PA-induced injury in Leydig cells.

The aim of this study was to evaluate the effects of curcumin on PA-induced injury in MLTC-1 cells and further explore the mechanism by which curcumin ameliorates cell apoptosis. Besides, we determined the impact of curcumin on testosterone levels in PA-exposed Leydig cells. Gaining a better understanding regarding the protective effects of curcumin and its mechanism of action against PA-induced injury in Leydig cells may be instrumental for the design of novel therapies for treating obesity-induced male infertility.

## Materials and methods

### Materials

Curcumin, TG, 4-PBA, ethylene diamine tetra acetic acid (EDTA) and dimethyl sulfoxide (DMSO) were procured from Sigma-Aldrich (St Louis, Missouri, USA). The murine Leydig tumor cell line MLTC-1 was obtained from Cell Institute of Shanghai, Chinese Academy of Sciences (Shanghai, China). Radioimmunoprecipitation assay (RIPA) lysis buffer, phenylmethylsulfonyl fluoride (PMSF), trypsin and Tris-buffered saline-Tween-20 (TBST) were purchased from Solarbio (Beijing, China). RPMI 1640 medium was purchased from Hyclone (Utah, USA). Fetal bovine serum (FBS) was procured from Gibco (Grand Island, New York, USA). Caspase 3 Activity Colorimetric Assay Kit, Total Protein Extraction Kit, and bicinchoninic acid (BCA) Protein Assay Kit were purchased from the Nanjing Jiancheng Bioengineering Institute (Nanjing, China). Cell Counting Kit 8 (CCK 8) and Annexin V-fluorescein isothiocyanate (FITC)/Propidium Iodide (PI) Apoptosis Analysis Kit were obtained from Beijing Zoman Biotechnology Co., Ltd. (Beijing, China). Testosterone ELISA Kit was purchased from Ji Yin Mei (Wuhan, China). Rabbit anti-mouse primary antibodies against BAX (sc-4239) and β-actin (sc-517,582) were obtained from Santa Cruz Biotechnology, Inc. (Santa Cruz, California, USA). Rabbit anti-mouse primary antibodies against CHOP (ab10444) and GRP78 (ab32618) were purchased from Abcam (Cambridge, UK). The goat anti-rabbit secondary antibodies were procured from Proteintech (Wuhan, China).

### Cell culture and treatment

**Fig. 1:**
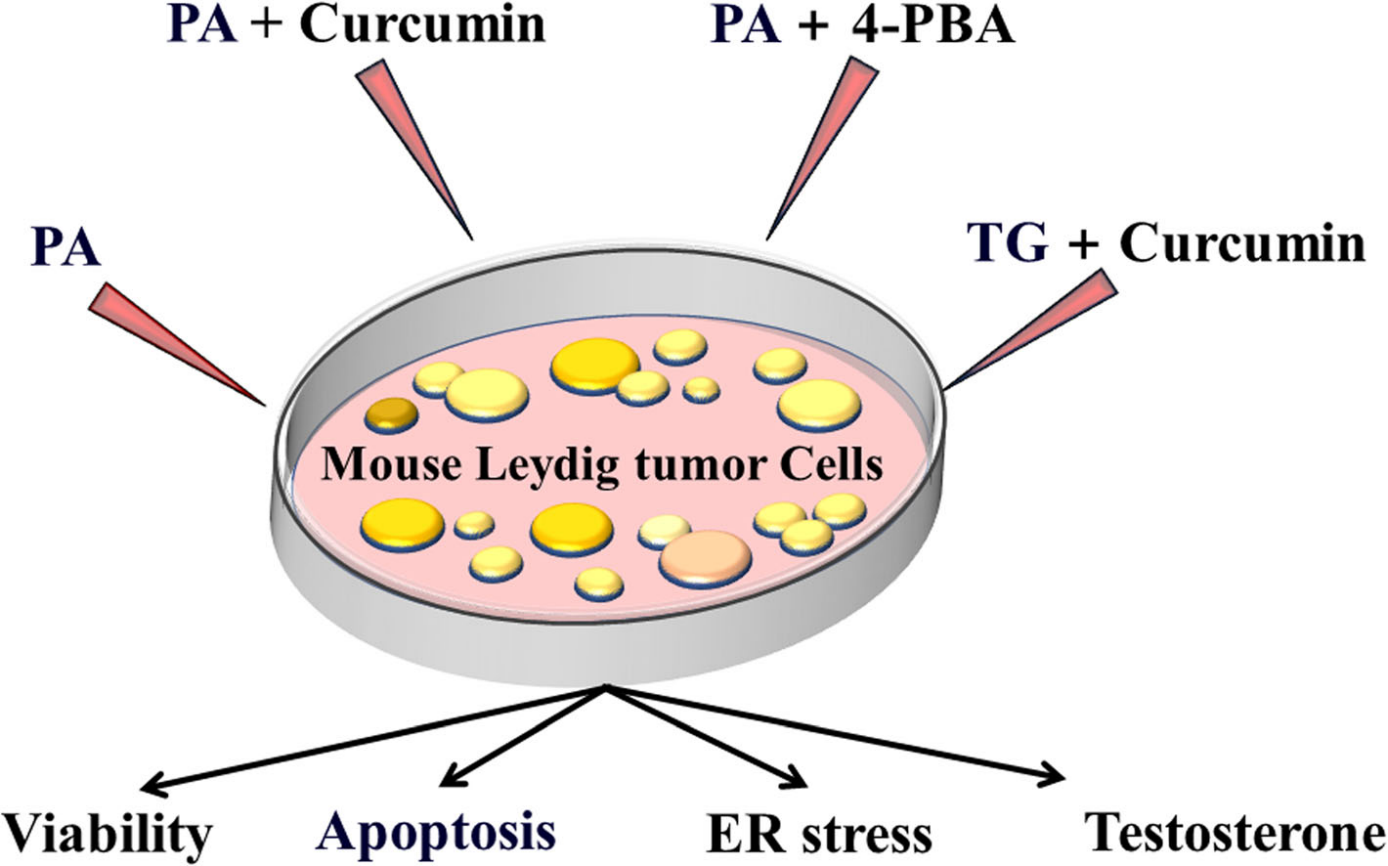
Schematic of the experimental design. MLTC-1 cells were treated with PA, PA + curcumin, PA + 4-PBA or TG + curcumin to explore the mechanism of curcumin that protects against PA induced cell apoptosis

In brief, MLTC-1 cells were cultured in RPMI 1640 medium supplemented with 10% FBS, penicillin (100 IU/mL), and streptomycin (100 μg/mL) and maintained at 37 °C in a humidified incubator containing 95% air and 5% CO_2_. Curcumin, 4-PBA, and TG were prepared in DMSO with a final DMSO concentration of no more than 0.1% (v/v). After reaching 70–80% confluency, cells were divided into four groups, treated with various concentrations of PA (0, 50, 100, 200, and 400 μM), PA (400 μM) + curcumin (0, 5, 10, 20, and 40 μM), PA (400 μM) + 4-PBA (500 nM), and TG (500 nM) + curcumin (20 μM), respectively, followed by incubation for 24 h (Fig. 1). Cells were then collected to detect viability, apoptosis, and caspase 3 activity, and used to perform western blot analysis (Fig. 1).

### Estimation of cell viability

To explore the toxic effects of PA on Leydig cells, MLTC-1 cells were treated with increasing concentrations of PA (0–400 μM), and cell viability was determined by CCK-8 assay according to the manufacturer’s instructions. Briefly, MLTC-1 cells were seeded at a density of 2 × 10^4^ cells per well in 96-well plates. After the incubation of cells with different treatments for 24 h, 10 μL of CCK-8 solution was added to each well. Cells were then incubated for 2 h at 37 °C, followed by measurement of absorbance at 405 nm using a microplate reader (Bio-Rad 680, CA, USA). All experiments were performed in triplicate.

### Cell apoptosis assay

After various treatments, MLTC-1 cells were washed with PBS, digested with 0.25% EDTA-free trypsin and harvested. Thereafter, cells were centrifuged at 500×g for 5 min, washed twice with cold PBS, and adjusted to a final concentration of 1 × 10^5^ cells/mL. Cells were initially re-suspended in 50 μL of binding buffer, after which 5 μL of PI was added, and the mixture was then incubated for 15 min at room temperature in the dark. Finally, 450 μL of binding buffer and 1 μL of Annexin V-FITC were added, and samples were further incubated for 15 min in the dark. Apoptosis was detected by flow cytometry (FACSCalibur™, BD Biosciences, CA, USA) within 1 h of the last incubation. Measurements for each sample were conducted in triplicate.

### Western blot analysis

After different treatments, cells were harvested and total protein was extracted using RIPA lysis buffer. Protein concentrations were then determined by BCA Protein Assay Kit. Each protein sample (30 μg) was separated on 12% sodium dodecyl sulfate (SDS)-polyacrylamide gels and then electrotransferred onto polyvinylidene fluoride (PVDF) membranes. Membranes were then blocked with 10% nonfat milk in TBST for 2 h and incubated overnight with anti-β-actin, anti-BAX, anti-CHOP, and anti-GRP78 primary antibodies at 4 °C. Thereafter, these blots were incubated with horseradish peroxidase-conjugated secondary antibodies (1:4000) for 30 min at 37 °C. The immunoreactive bands on membranes were visualized using SuperSignal West Pico Kit (Proteintech, Wuhan, China) and detected by Bio-Rad imaging system (Bio-Rad, CA, USA), according to the manufacturer’s instructions. Densitometric analysis was performed using ImageJ software 1.48 (Bethesda, MD, USA).

### Caspase 3 activity measurement

Caspase 3 activity was measured using Caspase-3 Activity Colorimetric Assay Kit according to the manufacturer’s instructions. After different treatments, MLTC-1 cells were harvested by centrifugation and incubated in lysis buffer on ice for 15 min. Cell lysates were then centrifuged at 15,000 rpm for 15 min at 4 °C. The protein content was determined using the BCA Protein Assay Kit. Sample aliquots were then incubated with the caspase 3 substrate in a microplate at 37 °C for 4 h and the absorbance was recorded at 405 nm using a microplate reader (Bio-Rad 680, CA, USA).

### Animal care and treatment

All the animal experiments performed in the present study were approved by the Committee for the Ethics on Animal Care and Experiments of Jiujiang University (approval No. SYXK(GAN)2019–0001). Male Sprague-Dawley rats (*n* = 40, body weight: 220–240 g) were obtained from the Experimental Animal Center of Jiujiang University. All rats were housed under conditions of controlled temperature (20–25 °C), humidity (50 ± 5%), and lighting (12 h light/12 h dark cycle) with free access to food and water. Curcumin was dissolved by olive oil and administered orally by oral gavage. The rats were randomly divided into four groups, including a control group (*n* = 10), HFD group (n = 10), HFD + curcumin group (n = 10) and a curcumin group (n = 10). All rats were fed ad libitum with an HFD for 4 weeks, with the exception of those in the curcumin and control groups. The rats in the curcumin group and HFD + curcumin group were orally administered curcumin (100 mg/kg/day) for 4 weeks, while those in the control and HFD groups were administered an identical volume of the vehicle. Following treatment, the rats were anaesthetized with an intraperitoneal injection of sodium pentobarbital (45 mg/kg), and blood samples were obtained from the abdominal aorta.

### Testosterone measurement

To evaluate the effects of curcumin on testosterone production in PA-treated MLTC-1 cells, cells were exposed to PA and/or curcumin, and testosterone levels were then determined in the culture medium using specific ELISA kits. In brief, the MLTC-1 cells were treated with PA (400 μM) and/or curcumin (20 μM), and co-incubated with 5 IU/mL hCG for 24 h, followed by measurement of testosterone concentration in cell-culture supernatants (100,000 cell/mL culture supernatant) by employing a testosterone ELISA kit according to the manufacturer’s instructions. Blood samples were obtained from the abdominal aorta. After centrifuging the samples at 1500 g and 4 °C for 10 min, the supernatant sera were obtained for further detection. Serum level of testosterone was measured using kits, according to the manufacturer’s instructions. The minimum detectable concentration of testosterone was 0.02 ng/mL. The intra- and inter-assay coefficients of variation were < 9 and < 15%, respectively. Assessment for each sample was carried out in triplicate.

### Statistical analysis

The data were analyzed by ANOVA, followed by Fisher’s least significant difference test and independent samples Student’s t test, with SPSS software, version 13.0 (SPSS, Chicago, IL, USA). All the data are presented as a mean ± standard error of the mean (SEM). For all analysis, *p* values of < 0.05 were considered statistically significant.

## Results

### PA induces apoptosis via the activation of ER stress in MLTC-1 cells

**Fig. 2:**
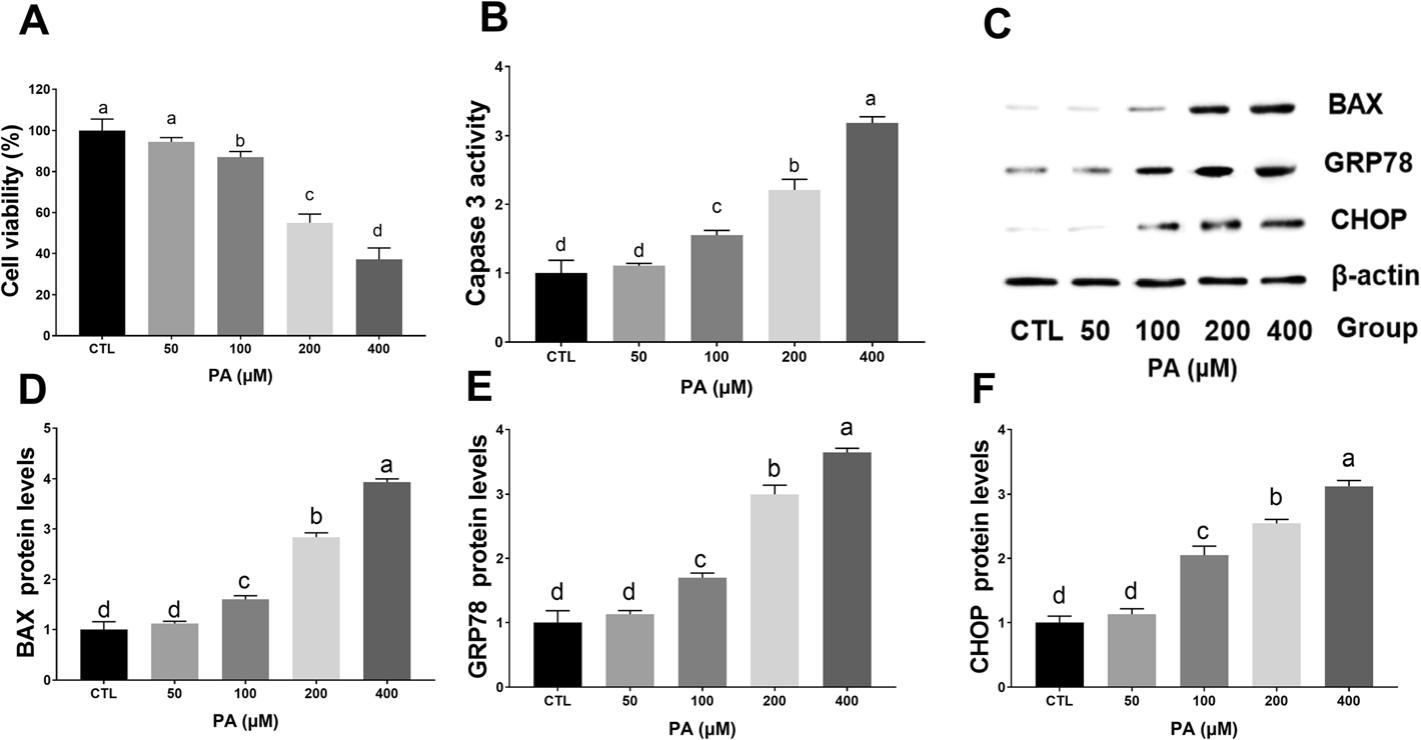
PA induces apoptosis and ER stress in MLTC-1 cells. Cells were treated with increasing concentrations of PA (50–400 μM) for 24 h, and cell viability was measured by CCK 8 assay (**a**). Caspase-3 activity was measured using the Caspase-3 Activity Colorimetric Assay (**b**). Western blot analysis (**c**) was performed to detect the relative expression of apoptosis-related BAX (**d**) and ER stress marker GRP78 (**e**) and CHOP (**f**). The proteins expression levels were normalized to β-actin. The statistical analysis results are shown in the bar graphs. The data are represented as the mean ± SEM of three independent experiments. Bars with different letters are significantly different (*p* < 0.05)

As indicated in Fig. 2a, PA (100–400 μM) significantly decreased cell viability. To investigate whether the decreased cell viability was due to the induction of apoptosis, PA-treated MLTC-1 cells were analyzed by flow cytometry and western blotting. The results demonstrated that treatment of cells with 100–400 μM PA increased the expression of apoptosis-related genes caspase 3 and BAX in a dose-dependent manner (Fig. 2b-d). After treating cells for 24 h with 50–400 μM PA, we determined the expression of ER stress marker genes (GRP78 and CHOP) by western blot analysis. The results demonstrated a dose-dependent increase in the expression of GRP78 and CHOP upon exposure to 100–400 μM PA (Fig. 2c, e, f). At 400 μM, the cell viability decreased to approximately 40%, and apoptosis-related genes and ER stress marker genes showed elevated expression. We therefore used 400 μM in the subsequent PA treatments.

### Curcumin attenuates PA-induced cytotoxicity and ER stress in MLTC-1 cells

**Fig. 3:**
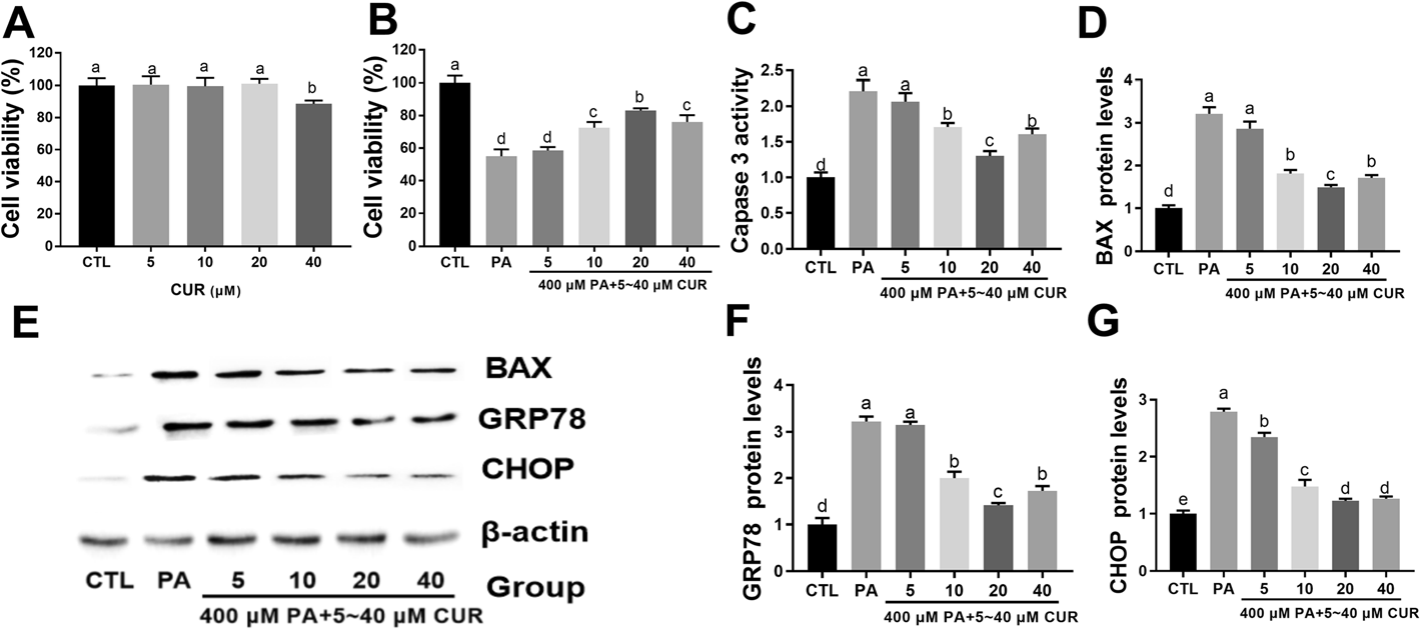
Curcumin attenuates the PA-induced apoptosis and ER stress in MLTC-1 cells. Cells were treated with different concentrations of curcumin (5–40 μM) in the absence (**a**) and presence (**b**) of PA (400 μM) for 24 h and then processed for cell activity analysis CCK 8 assay. Caspase-3 activity was measured using the Caspase-3 Activity Colorimetric Assay (**c**). Western blot analysis (**e**) was performed to detect the relative expression of apoptosis-related BAX (**d**) and ER stress marker GRP78 (**f**) and CHOP (**g**). The proteins expression levels were normalized to β-actin. The statistical analysis results are shown in the bar graphs. The data are represented as the mean ± SEM of three independent experiments. Bars with different letters are significantly different (*p* < 0.05)

To determine the effect of curcumin on Leydig cell viability, MLTC-1 cells were treated with different curcumin concentrations (5–40 μM) for 24 h. As shown in Fig. 3a, curcumin concentrations up to 20 μM did not alter the viability of MLTC-1 cells, whereas 40 μM curcumin significantly decreased cell viability compared to that of the control group cells. Furthermore, reduced cell viability due to treatment with 400 μM PA was observed to be restored in response to 20 μM curcumin (Fig. 3b). Meanwhile, 20 μM curcumin was shown to effectively reduce caspase 3 activity and the expression of BAX protein (Fig. 3c-e). In addition, 400 μM PA-induced increase in GRP78 and CHOP expression was inhibited by 20 μM curcumin (Fig. 3e-g). Interestingly, we observed that the 40 μM curcumin group had lower cell viability than the 20 μM group and higher viability than the PA-treated group (Fig. 3b). Similarly, the 40 μM curcumin group had higher caspase 3 activity and expression of BAX and GRP78 than the 20 μM group (Fig. 3c-f). However, no significant difference was observed in the CHOP expression between the 20 μM group and 40 μM group (Fig. 3e, g).

### 4-PBA attenuates PA-induced cytotoxicity, apoptosis, and ER stress in MLTC-1 cells

**Fig. 4:**
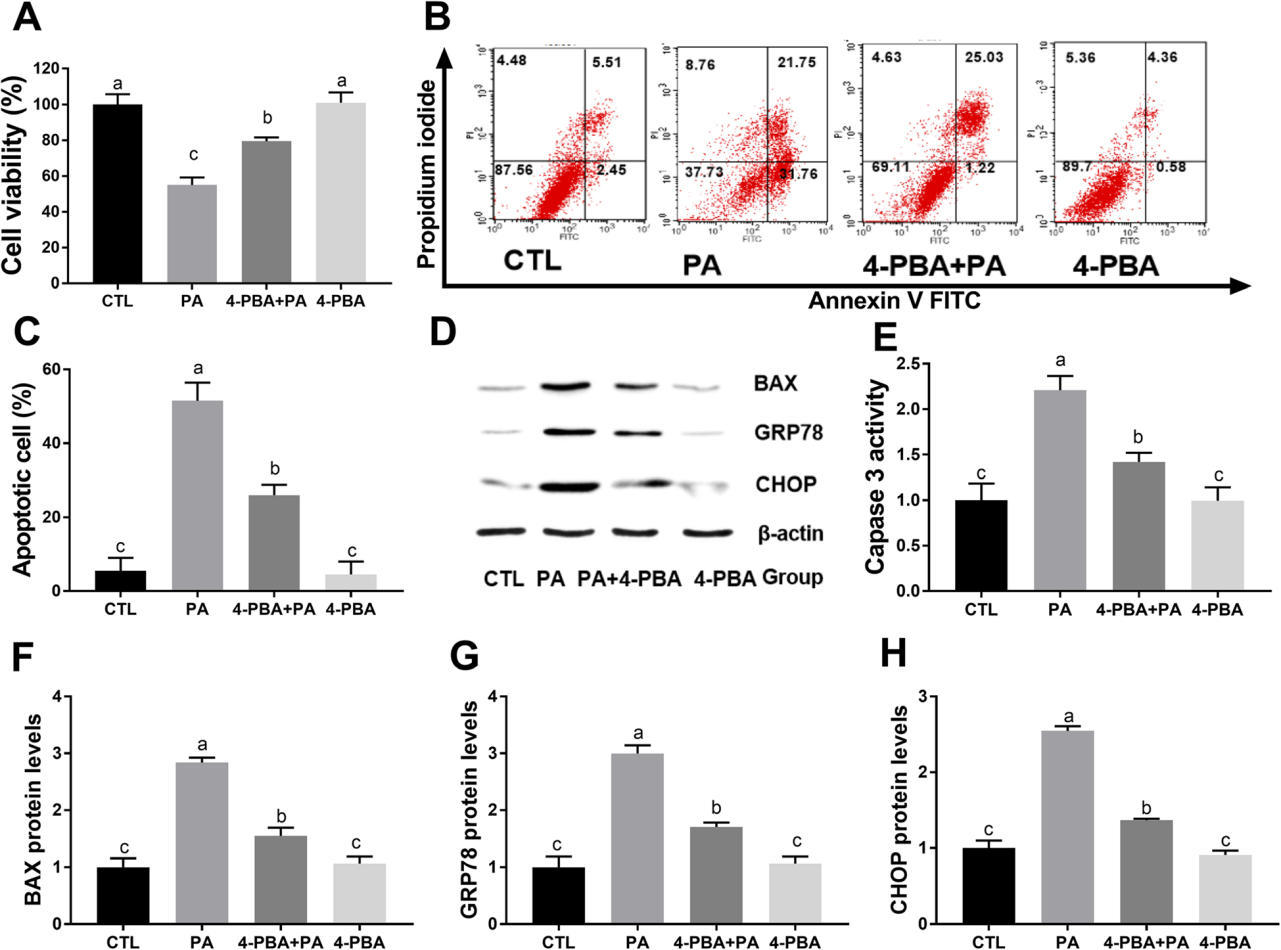
4-PBA attenuates the PA-induced apoptosis and ER stress in MLTC-1 cells. Cells were treated with PA (400 μM) in the absence and presence of 4-PBA (500 nM) for 24 h and then processed for cell activity analysis CCK 8 assay (**a**). Apoptosis analysis was detected by flow cytometry (**b**, **c**). Western blot of BAX, GRP78, and CHOP expression are shown (**d**). The Caspase 3 activity of the MLTC-1 cells is shown (**e**). The relative BAX expression (**f**), GRP78 expression (**g**), and CHOP expression (**h**) are depicted. The proteins expression levels were normalized to β-actin. The statistical analysis results are shown in the bar graphs. The data are represented as the mean ± SEM of three independent experiments. Bars with different letters are significantly different (*p* < 0.05)

The CCK 8 assay and flow cytometric analysis revealed that PA-exposure significantly reduced cell viability and induced apoptosis, while treatment with 4-PBA effectively restored cell viability and inhibited apoptosis (Fig. 4a-c). In addition, it was observed that 4-PBA treatment markedly reduced caspase 3 activity and the expression of BAX, GRP78, and CHOP in the PA-treated MLTC-1 cells (Fig. 4d-h).

### Curcumin protects MLTC-1 cells against TG-induced cytotoxicity, ER stress, and apoptosis in MLTC-1 cells

**Fig. 5:**
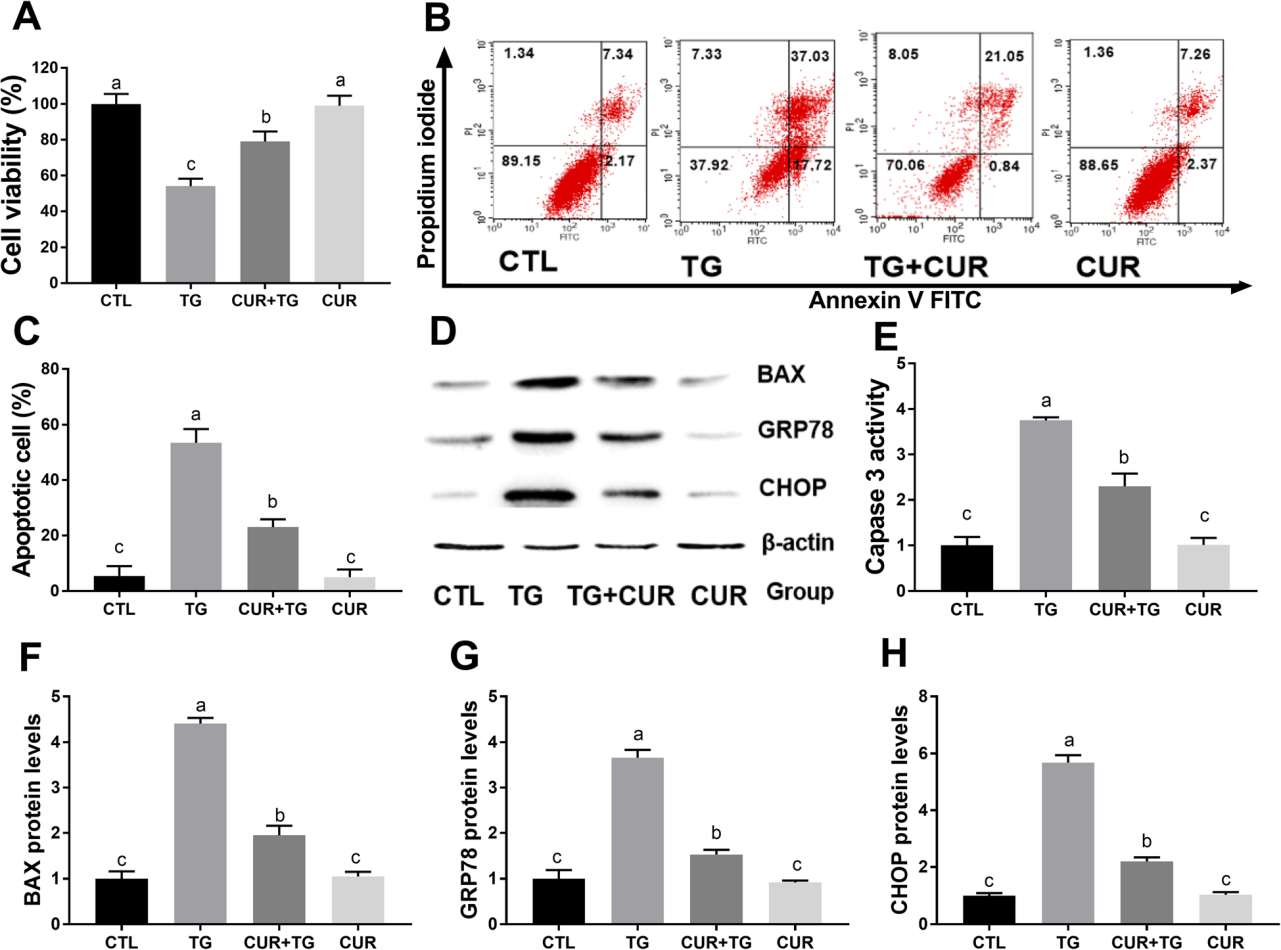
Curcumin attenuates the TG-induced apoptosis and ER stress in MLTC-1 cells. Cells were treated with TG (500 nM) in the absence and presence of curcumin (20 μM) for 24 h and then processed for cell activity analysis CCK 8 assay (**a**). Apoptosis analysis was detected by flow cytometry (**b**, **c**). Western blot of BAX, GRP78, and CHOP expression are shown (**d**). The Caspase 3 activity of the MLTC-1 cells is shown (**e**). The relative BAX expression (**f**), GRP78 expression (**g**), and CHOP expression (**h**) are depicted. The proteins expression levels were normalized to β-actin. The statistical analysis results are shown in the bar graphs. The data are represented as the mean ± SEM of three independent experiments. Bars with different letters are significantly different (*p* < 0.05)

As expected, TG induced reduction in MLTC-1 cell viability, increased ER stress markers GRP78 and CHOP, and activated caspase 3 and BAX, while treating cells with 20 μM curcumin significantly restored viability and inhibited TG-induced ER stress and apoptosis (Fig. 5).

### Curcumin restores PA-mediated inhibition of testosterone secretion in MLTC-1 cells

**Fig. 6:**
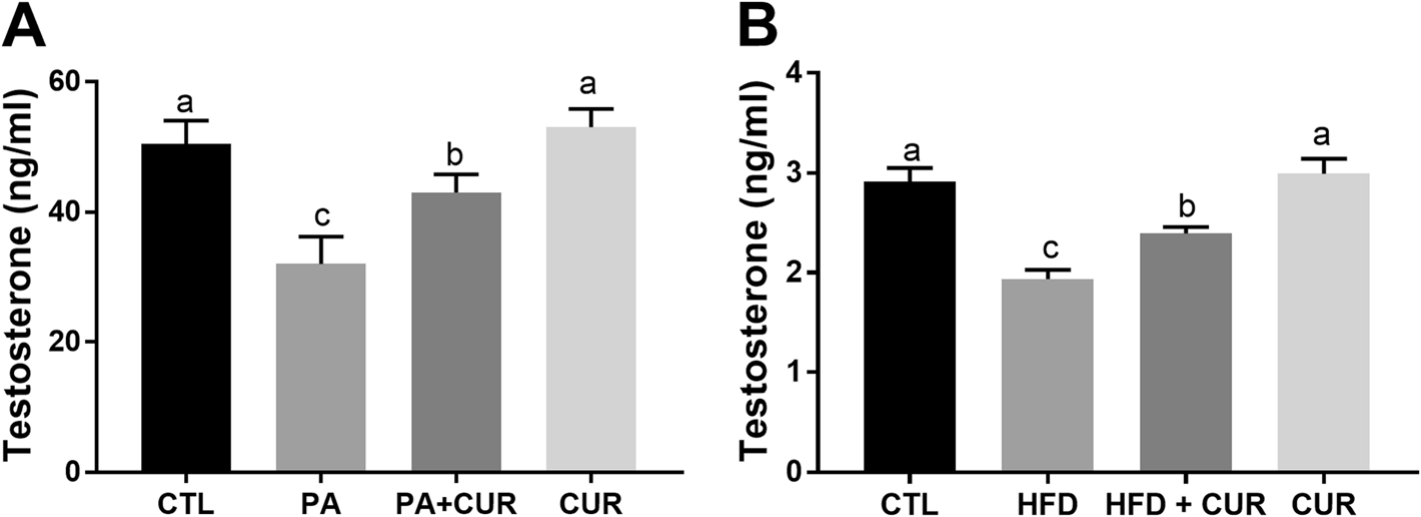
Effects of curcumin on testosterone generation in PA-treated MLTC-1 cells. MLTC-1 cells were treated with hCG for 24 h and the concentration of testosterone in the culture supernatants was then measured employing the ELISA assay (**a**). The rats were orally administered curcumin for 4 weeks, and serum level of testosterone was measured using the ELISA assay (**b**). The statistical analysis results are shown in the bar graphs. Data are presented as the mean ± SEM of three independent experiments. Bars with different letters are significantly different (*p* < 0.05)

Testosterone secretion was stimulated in MLTC-1 cells by the addition of hCG to PA-containing culture medium in which cells were incubated for 24 h. As shown in Fig. 6a, in 400 μM PA-treated cells, concentration of testosterone was significantly decreased compared to that in untreated control cells, whereas curcumin significantly attenuated the reduction of testosterone secretion in MLTC-1 cells exposed to PA. Subsequently, we explored the effect of curcumin on testosterone production in HFD-fed rats. We found that rats fed an HFD exhibited abnormal serum hormone levels, manifested as reduced serum levels of testosterone. Serum testosterone levels were observed to be restored following treatment with curcumin (Fig. 6b).

## Discussion

Curcumin, a natural diphenolic compound, possesses numerous health beneficial effects such as anti-inflammatory, anti-obesity, and antioxidant properties [15–19]. Due to its pharmacological efficacy, curcumin has been studied widely in various research areas. In this work, we demonstrated that PA reduced Leydig cell viability, activated caspase 3, and enhanced the expression levels of apoptosis-related protein BAX and ER stress markers GRP78 and CHOP. Curcumin significantly suppressed PA-induced decrease in cell viability and the expression levels of apoptosis-related protein and ER stress markers. In addition, curcumin could restore PA- or HFD-mediated inhibition of testosterone secretion by Leydig cells in vitro and in vivo.

The increase in the male infertility rates has been reported to be in parallel with the increasing rates of obesity [23]. Studies have shown that increased weight in men is correlated with lower testosterone levels and reduced fertility. Moreover, the odds of infertility have been reported to increase by approximately 10% for every 9 kg (20 pounds) a man is overweight [23, 24]. In addition to reduced plasma testosterone levels, obesity has also been associated with chronic elevation of plasma FFAs [9]. PA is a common type of saturated FFA and is known to increase in obese individuals. Recently, PA has been reported to cause toxicity in various types of cells, including Leydig cells. PA-mediated cytotoxicity was observed to occur via reduction of cell viability and induction of apoptosis [9, 25–28]. However, the molecular mechanism underlying PA-induced apoptosis in Leydig cells is still not well understood. To investigate the possible PA-induced apoptotic mechanisms in Leydig cells, we first determined the cytotoxicity of PA in MLTC-1 cells. In the present study, PA was found to activate caspase 3 and upregulate BAX protein expression in a dose-dependent manner in order to induce apoptosis in MLTC-1 cells*,* which is in agreement with a previous study [9]. Earlier reports have suggested that PA induces apoptosis in different types of cells by activating ER stress*-*mediated apoptotic pathways [27, 29, 30]. To confirm the role of ER stress in PA-induced MLTC-1 cell apoptosis, we checked the expression of ER stress marker genes GRP78 and CHOP. Our data showed a significant dose-dependent increase in the expression of GRP78 and CHOP proteins in PA-treated MLTC-1 cells. These results indicate that inhibition of ER stress in Leydig cells might effectively ameliorate the toxic effects of PA.

Curcumin is a phenolic compound that has been shown to play a significant protective role in inhibition of ER stress-mediated apoptosis [20, 31, 32]. In the current study, MLTC-1 cells were used as a suitable model to investigate the effects of curcumin on PA-induced cell injury and its underlying protective mechanism. Initially, the effects of curcumin on the viability and apoptosis of MLTC-1 cells were examined. Our data indicated that low concentrations of curcumin (5–20 μM) ameliorated the PA-induced decrease in cell viability and reduced the expression of apoptosis-associated genes, including BAX and caspase 3, which is consistent with a previous study [22]. Based on these results, it was concluded that curcumin inhibits PA-induced apoptosis in Leydig cells. Interestingly, in this study, curcumin at 40 μM was observed to show a negative impact on cell viability, indicating that curcumin may have cytotoxic effect at certain high concentrations. Although the reason for this cytotoxic effect has never been fully elucidated, we suspect that an exposure time of 24 h may be long enough for 40 μM curcumin to accumulate in MLTC-1 cells to cause apparent cell damage.

In this study, we have used 400 μM PA and 20 μM curcumin, which seem to be higher than that in vivo, to perform in vitro experiments. In fact, PA and curcumin can achieve 20 and 400 μM in vivo by oral supplementation, respectively. Although curcumin’s low oral bioavailability limited its application, many efforts have been carried out to improve its solubility and oral bioavailability in the past years. Nowadays, it has been transformed into a variety of different formulations, peak plasma concentration of curcumin in vivo is about 50–20,000 ng/mL depending on the formulation [33–39]. The physiological concentration of PA in vivo is about 100 μmol/L. Interestingly, in obese patients, plasmatic PA concentration rises up to three- to fivefold when compared with matched healthy subjects [40–42].

Curcumin was reported to inhibit ER stress caused by cerebral ischemia-reperfusion injury in rats [32]. Increased apoptosis was observed in the hearts of diabetic mice, which was attenuated by curcumin, ultimately improving cardiac function [43]. In the current study, our data revealed that the PA-induced increase in GRP78 and CHOP protein expression was suppressed by curcumin. These data indicate that curcumin might attenuate PA-induced cytotoxicity and ER stress in MLTC-1 cells. Additionally, to check the involvement of ER stress in apoptosis induced by PA, 4-PBA was used as an ER stress inhibitor, and as expected, it was observed that inhibition of ER stress by 4-PBA attenuated the PA-induced cell apoptosis. However, curcumin was shown to protect MLTC-1 cells against reduction of viability, apoptosis and ER stress caused by the ER stress inducer TG. These results further confirmed that ER stress was indeed involved in PA-induced MLTC-1 cell apoptosis and that curcumin can indeed protect Leydig cells against TG- or PA-induced damage through inhibition of ER stress response.

Realizing the importance of Leydig cells in testosterone secretion, we subsequently explored the effects of PA and/or curcumin on testosterone secretion in MLTC-1 cells. As expected, PA treatment significantly decreased the production of testosterone in MLTC-1 cells, while curcumin restored testosterone levels effectively. We then investigated the effects of curcumin on testosterone production in diet-induced obesity male rats. Rats fed an HFD were treated with or without curcumin for 4 weeks. We found that the testosterone levels were decreased in rats fed an HFD, and treatment with curcumin upregulated the decreased levels of serum testosterone. The results of the present study suggested that curcumin treatment may ameliorate diet-induced reduction of testosterone levels. Similar to this, an earlier study showed that curcumin ameliorated HFD-induced decrease in serum testosterone and reduced HFD-induced spermatogenesis dysfunction and apoptosis [16]. These findings suggest that PA may play an important role in male infertility and that curcumin can be used as a promising therapeutic agent for treating obesity-associated male infertility.

A critical factor considered to affect serum and/or intratesticular testosterone levels is the number of Leydig cells. Physiologically, in a normal testis, a certain degree of apoptosis can be observed, which plays an important role in discarding decrepit and abnormal cells, thereby maintaining the population of Leydig cells and testosterone levels [44]. Excessive Leydig cell apoptosis caused by testis impairment could result in decreased testosterone levels, leading to apoptosis of spermatogenic cells, causing infertility. Interestingly, in a study by Mu et al., excessive activation of autophagy was observed in sperm samples from obese male patients, and inhibition of autophagy was observed to improve the decreased fertility of obese male mice [8]. Therefore, in the present study, PA was shown to decrease the number of MLTC-1 cells and compromise the normal endocrine function of these cells, thereby suppressing the secretion of testosterone. Although curcumin restored testosterone production in PA-treated MLTC-1 cells, the precise mechanism through which it regulates the synthesis of testosterone in Leydig cells remains unclear and needs further investigation.

## Conclusions

In summary, our results suggest that PA induces apoptosis in testicular Leydig cells through the ER stress signaling pathway. Furthermore, curcumin could potentially protect these cells from PA-induced apoptosis and restored testosterone production. These findings reflect the potential of natural compounds in the development of future therapeutic approaches for the treatment of obesity-related male infertility.

## Data Availability

The datasets used and/or analyzed during the current study available from the corresponding author on reasonable request.

## Abbreviations

4-PBA: 4-phenylbutyric acid
BAX: B-cell lymphoma-2 (BCL-2) associated X protein
BCA: Bicinchoninic acid
CHOP: CCAAT/enhancer binding protein homologous protein
CUR: Curcumin
DMSO: Dimethyl sulfoxide
EDTA: Ethylene diamine tetra acetic acid
ELISA: Enzyme-linked immunosorbent assay
ER: Endoplasmic reticulum
FBS: Fetal bovine serum
FFAs: Free fatty acids
FITC: Fluorescein isothiocyanate
GRP78: Glucose-regulated protein 78
hCG: Human chorionic gonadotropin
HFD: High-fat diet
LH: Luteinizing hormone
MLTC-1: Murine Leydig tumor cell line 1
PA: Palmitic acid
PI: Propidium iodide
PMSF: Phenylmethylsulfonyl fluoride
RIPA: Radioimmunoprecipitation assay
RPMI 1640: Roswell Park Institute-1640
SDS: Sodium dodecyl sulfate
TBST: Tris-buffered saline-Tween-20
TG: Thapsigargin
